# Extended TAM based acceptance of AI-Powered ChatGPT for supporting metacognitive self-regulated learning in education: A mixed-methods study

**DOI:** 10.1016/j.heliyon.2024.e29317

**Published:** 2024-04-09

**Authors:** Nisar Ahmed Dahri, Noraffandy Yahaya, Waleed Mugahed Al-Rahmi, Ahmed Aldraiweesh, Uthman Alturki, Sultan Almutairy, Anna Shutaleva, Rahim Bux Soomro

**Affiliations:** a Faculty of Social Sciences and Humanities, School of Education, University Teknologi Malaysia, UTM Sukadi, Johor, 81310, Malaysia; b Educational Technology Department, College of Education, King Saud University, P.O. Box 21501, Riyadh, 11485, Saudi Arabia; c Ural Federal University Named After the First President of Russia B. N. Yeltsin, 620002, Ekaterinburg, Russia; d Institute of Business Administration, Shah Abdul Latif University, Khairpur, Pakistan

**Keywords:** Artificial intelligence, AI in education, Metacognition, Self-regulated learning, ChatGPT, Technology acceptance model

## Abstract

This mixed-method study explores the acceptance of ChatGPT as a tool for Metacognitive Self-Regulated Learning (MSRL) among academics. Despite the growing attention towards ChatGPT as a metacognitive learning tool, there is a need for a comprehensive understanding of the factors influencing its acceptance in academic settings. Engaging 300 preservice teachers through a ChatGPT-based scenario learning activity and utilizing convenience sampling, this study administered a questionnaire based on the proposed Technology Acceptance Model at UTM University's School of Education. Structural equation modelling was applied to analyze participants' perspectives on ChatGPT, considering factors like MSRL's impact on usage intention. Post-reflection sessions, semi-structured interviews, and record analysis were conducted to gather results. Findings indicate a high acceptance of ChatGPT, significantly influenced by personal competency, social influence, perceived AI usefulness, enjoyment, trust, AI intelligence, positive attitude, and metacognitive self-regulated learning. Interviews and record analysis suggest that academics view ChatGPT positively as an educational tool, seeing it as a solution to challenges in teaching and learning processes. The study highlights ChatGPT's potential to enhance MSRL and holds implications for teacher education and AI integration in educational settings.

## 1 Introduction

Metacognitive Self-Regulated Learning (SRL) is a crucial component in fostering successful academic performance and the achievement of learning objectives [1]. This multifaceted approach empowers students to manage their learning processes, effectively guiding their educational journey [2]. In pre-service teacher education, the cultivation of metacognitive SRL has a significant impact [3]. Preparing future educators is not solely about imparting subject matter knowledge but equipping them with the necessary skills to adapt and excel in a rapidly evolving digital learning environment [4]. Metacognitive SRL is not only a supporter of teachers as learners but also enables them to better serve their students by modeling effective learning strategies [5]. This is particularly critical, as teachers are the architects of the educational experience, and their proficiency in leveraging metacognitive SRL techniques can significantly enhance the quality of instruction, ultimately benefitting the entire educational ecosystem [3]. Thus, understanding the essence of metacognitive SRL and its application in pre-service teacher education is paramount in shaping a cohort of educators equipped to navigate and excel in the online learning landscape. Jiang et al. [3] advocated for the use of "teaching with metacognition" as a means to enhance instructional effectiveness. Additionally [3], emphasized that understanding teachers' self-awareness about their teaching should be the foundation for improving teacher professional development. However, the absence of suitable metrics has hindered research into teacher metacognition [3]. Metacognition holds significant importance in education, particularly in learning, teaching, and teacher training [3,6]. It involves understanding and regulating one's thinking, which can lead to better learning [7]. In recent years, it's been widely recognized as essential for successful learning [8] and effective teaching [9]. Metacognition is an essential skill for pre-service teachers since it shapes their future pedagogical practices, and they are crucial in molding the next generation of learners. In essence, the underlying premise posits the significance of metacognition and self-regulated learning (SRL) in the context of education [6]. Numerous studies have consistently demonstrated the substantial influence of SRL, with a specific emphasis on metacognition, on students' academic performance, irrespective of their innate abilities or prior achievements [[10], [11], [12]].

Self-regulation pertains to the degree of awareness that learners possess regarding their strengths and weaknesses, their ability to employ effective learning strategies, self-motivation for active engagement in learning, and their capacity to formulate and implement strategies and tactics to enhance the learning process [[13], [14], [15]].

Successful online learners set specific goals, focus on learning rather than performance, and possess task-specific self-efficacy and self-instruction tendencies, which are crucial in learning [15,16]. On the other hand, metacognition focuses specifically on how learners can actively oversee and purposefully guide their learning endeavors [10,11]. The concept of self-regulated learning is grounded on the principle that students should shoulder responsibility for their learning and actively participate in the learning process [17]. According to Schraw et al. [18], metacognition's pivotal role is enabling individuals to effectively monitor their current knowledge and skill levels, optimize the allocation of limited learning resources, and assess their ongoing state of learning. Empirical support for this concept is evident in Dent and Koenka's [19] meta-analysis of 61 studies, where measures of metacognitive processes exhibited stronger correlations with achievement compared to measures of cognitive strategy utilization. This suggests that the decision of when to employ different cognitive strategies may hold more significance than the frequency of their utilization. Likewise, SRL, metacognition has two basic components: 1) Metacognitive knowledge represents what a learner understands about their learning processes or how to engage effectively with specific tasks, 2) Metacognitive skills involve the capacity to manage these activities. Proficient use of metacognitive skills relies on applying metacognitive knowledge, encompassing students' ability to assess their progress in cognitive tasks and employ strategies to regulate their progress systematically [10,11]. Effective metacognitive learners exhibit a range of behaviours that encompass self-evaluation, record-keeping, monitoring of learning progress, seeking assistance from adults, verbalizing thoughts, goal setting, time management, engaging in peer learning, demonstrating resilience, mitigating distractions, seeking external resources, applying self-rewards or penalties based on outcomes, memorization, rehearsal of information, and awareness of personal weaknesses [20,21]. Effective learners employ various strategies, including setting specific proximal goals, adopting potent strategies for goal attainment, ongoing performance monitoring, modifying their physical and social environments to align with their goals, efficient time management, self-evaluation of methods, attributing causality to results, and adjusting future approaches [14].

Research on students with learning difficulties or lower academic attainment generally indicates lower self-regulated learning and metacognition [9,20,21]. Some interventions, such as self-regulated strategy development, have demonstrated the potential to improve academic skills in students with learning difficulties [22]. Certain studies indicate that girls tend to outperform boys in self-regulated learning, a trend consistent across different educational phases and countries [10,11,21]. Metacognitive self-regulated learning is critical for academic performance, as evidenced by studies [3,9,15]. Students with efficient metacognitive techniques can track their understanding, make objectives, and adjust to new learning situations.

However, employing artificial intelligence technologies to promote metacognitive self-regulated learning is a developing field of study. In our rapidly evolving world, educational institutions face the challenge of imparting every skill required for the future. Therefore, the focal point should shift from mere knowledge transmission to instructing students on "how to learn”. It is beneficial to nurture metacognitive and self-learning skills through education [8]. Recognizing their pivotal role, the revised Bloom's taxonomy has incorporated metacognitive skills across various educational levels [23].

A notable breakthrough in Artificial Intelligence (AI) is the emergence of Large Language Models (LLMs) like GPT. Initially designed for text-based tasks, these models can generate coherent, human-like narratives, sometimes surpassing human performance. Their effectiveness in various cognitive assessments is equally praiseworthy [24]. Furthermore, LLMs have evolved beyond text and can now interpret and generate content across multiple formats. their impact is already being felt in the fields of education and research [8,25]. The discourse concerning AI's cognitive capabilities continues to progress [26]. While some evaluations indicate that LLMs, including ChatGPT, exhibit attributes like context awareness, problem-solving, and reasoning [27], AI has not yet attained the complexity of human cognition. Nevertheless, the primary objective of AI is not to surpass human capabilities but to enhance them. By incorporating generative AI tools based on LLMs in educational contexts, educators can design tailored learning experiences that encourage metacognitive reflection and consistently promote the development of critical thinking skills [24]. Integrating Artificial Intelligence (AI) holds great promise for enhancing metacognitive self-regulated learning [2,28,29]. However, students must possess robust prior knowledge, cognitive skills, metacognitive skills, and motivation to resist distractions for effective engagement in these settings [2]. AI in education can further enhance metacognitive skills through scaffolding mechanisms, which support learners in specific tasks. These scaffolds promote self-awareness and reflection on learning strategies [30]. Generative AI solutions like ChatGPT, often known as educational chatbots or conversational agents, promise students personalized and interactive learning experiences in the ever-changing world of education [30]. It's gained attention in creative writing, coding, teaching innovation, sentiment analysis, and annotation tasks [31]. Research extensively explores ChatGPT's applications across academic fields. Khan et al. highlight its contributions to medicine and public health education [30]. Language learning enhances interactions and aids in vocabulary, grammar, and translation [30,31]. It's also useful in software education, identifying coding issues, and in economics and finance research for simulations [32].ChatGPT enhances active learning by bolstering learners' self-regulation. As defined by Pintrich [33], self-regulation involves learners' ability to control, monitor, and manage their learning processes independently to attain their educational objectives. Multiple studies emphasize the significance of learning motivation, engagement, and self-efficacy (SE) in self-regulation [28,34].

Additionally, ChatGPT can assist in creating interactive learning activities and recommending relevant learning materials such as articles, videos, and quizzes, allowing students to progress at their own pace. These activities and resources encourage students to apply their knowledge and critical thinking skills, promoting deeper engagement in the learning process. Alternatively, ChatGPT can serve as a cognitive tool to help learners organize and structure their knowledge [34,33]. These features collectively contribute to sustaining learners' engagement during the performance phase.

Following Zimmerman's [17] self-regulated learning (SRL) model, the learning process consists of three phases: forethought, performance, and self-reflection. In the forethought phase, learners assess learning tasks, establish goals, and formulate strategies. Engaging with ChatGPT enables learners to articulate their objectives, define the content they wish to explore and set milestones for their learning journey, empowering them to take charge of their learning during this initial phase. During the performance phase, learners are tasked with actively participating in learning activities and fine-tuning their learning approaches to achieve their objectives [17,35,36]. Learners benefit from actively posing and addressing questions pertinent to their studies to reach their desired learning outcomes [[37], [38]]. In response to students' academic queries, ChatGPT can furnish personalized feedback to enhance the learning experience [34,33]. This personalized feedback on assignments, essays, or projects aids learners in recognizing areas for improvement, alleviating potential discomfort that might arise from receiving direct and critical instructor feedback. In the self-reflection phase, learners evaluate their performance and provide insights into task outcomes and effectiveness [32,33]. ChatGPT provides tailored guidance and suggestions to support learners in this phase, completing the learning cycle.

Tayan et al. [39] critically examine the integration of AI chatbots like ChatGPT into higher education technology courses. They highlight the potential benefits of personalized learning and student engagement, such as tailored feedback and self-regulated learning prompts. Further research highlights the greater impact of ChatGPT across learning outcomes and teaching practices. Song and Song explore the intriguing question: can ChatGPT, an AI tool, boost EFL students' academic writing skills and motivation? ChatGPT significantly improved writing performance, with students crafting more accurate, fluent, and complex texts. Notably, motivation for writing tasks also soared, fueled by increased confidence and enjoyment. Students appreciated ChatGPT's personalized feedback and support, acting as a helpful scaffold for identifying errors and honing writing strategies [40]. Lin [41] investigates the use of ChatGPT as a virtual tutor for self-directed learning (SDL) among adult learners in asynchronous online contexts. ChatGPT facilitates collaboration and networking among educators, enabling participation in learning communities for idea exchange and professional development. It also supports writing instruction and feedback, providing educators with additional assistance. In the context of SDL, ChatGPT promotes independent learning by ensuring continuous access to resources [42]. Kok Ming and Mansor [43] investigate the potential of ChatGPT, an AI-powered language tool, in supporting teachers' professional development. The study highlights that ChatGPT's versatility holds promise for improving pedagogy through personalized learning paths, automated feedback, reflection prompts, and collaborative knowledge creation.

Further, Lodge et al. [44] explore the transformative potential of generative AI, such as ChatGPT, in education, proposing a "co-regulation" model challenging traditional student-teacher dynamics; AI's role includes providing personalized feedback, suggesting resources, and fostering metacognitive reflection. Wang and Lin [8] investigate the potential of AI as a tool for analyzing and supporting self-regulated learning (SRL). The study highlights the positive impact of AI on SRL.

The use of AI tools in pre-service teacher education can be advantageous for pursuing a career in teaching, especially, ChatGPT is a generative AI tool created by OpenAI that has attracted a lot of attention due to its ability to give students real-time feedback, explanations, and advice [1,24,27,40]. With the introduction of AI technology into educational settings, chances for automated assessment, personalized learning, and creative pedagogy have grown [45]. Artificial intelligence (AI) systems like ChatGPT have shown promise in improving metacognitive self-regulated learning [8,24,26]. According to Jin et al. (2023), they provide metacognitive processes with personalized guidance, relevant conversational engagements, and real-time feedback [1]. A limited amount of research has focused on pre-service teachers, a distinct population with particular expectations and characteristics, despite numerous studies examining educators' and students' acceptance of technology [42,43].

An emerging field of study is how pre-service teachers perceive AI tools, especially regarding metacognitive self-regulated learning. Nonetheless, not much study has been done on pre-service teachers' preparation for and adoption of ChatGPT in the context for metacognitive self-regulated learning, especially in regard to their ability to improve metacognitive self-regulated learning. Educators and organizations looking to use AI in teacher training programs must comprehend the aspects impacting pre-service teachers' acceptance of ChatGPT for metacognitive self-regulated learning. Although AI is becoming increasingly common in education [24,26,29], it is unclear how pre-service instructors in higher education perceive these tools and how effective they are. This study provides a significant gap in the literature by analyzing the factors influencing pre-service teachers' decision to use ChatGPT and examining their adoption of the AI tool for metacognitive self-regulated learning [10,28]. The study aims to determine pre-service teachers' adoption of ChatGPT for metacognitive self-regulated learning within the context of higher education.

• To investigate ChatGPT's acceptance in relation to metacognitive self-regulated learning.
• To examine the factors influencing the pre-service teachers' motivations for using ChatGPT for metacognitive activities.
• To comprehend pre-service teachers' use of ChatGPT for self-regulated and metacognitive learning through reflections and record analysis.

## 2 Theoretical framework

**Fig. 1:**
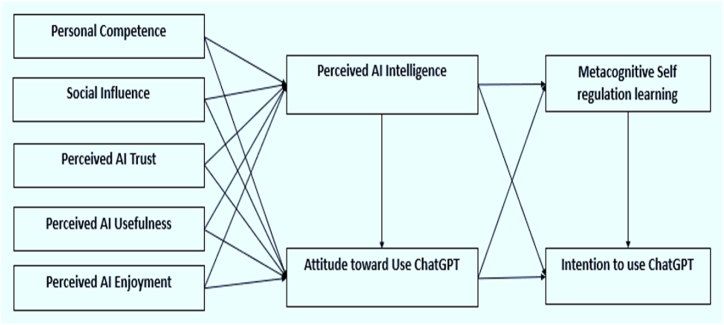
Proposed Metacognition Self-regulated learning acceptance model.

The study has selected the "Technology Acceptance Model" (TAM) as the fundamental framework for the adoption of ChatGPT [46], an AI tool for supporting metacognitive self-regulated learning. There are various reasons for selecting TAM for this study. First, TAM is ideally suited for analyzing AI adoption within the framework of metacognitive self-regulated learning, having been originally designed to forecast and explain consumer acceptance and usage of information technology [[45], [47], [48], [49]]. TAM has been extensively employed to understand the determinants of technology acceptance in various regions including Germany [50], US [51], Saudi Arabia [52], Pakistan [53], Malaysia [54], Turkey [55], Greece [56], Indonesia [57], South Korea [58], and China [56,57], and across different technology-related domains such as e-learning, remote education, massive open online courses, social media and mobile library applications demonstrating its robustness and usefulness [34,51,58]. TAM, originating from the theory of reasoned action, posits that an individual's behaviour is influenced by their behavioral intention (BI), with BI determined by subjective norms and attitudes [59]. Davis (1989) introduced TAM with two central factors: Perceived Usefulness (PU) and Perceived Ease of Use (PEOU) [49]. The model explains that PU and PEOU shape individuals' attitudes and both PU and attitudes toward technology usage predict BI [46,60]. It is particularly suitable for analyzing how different online learning methods are implemented [61]. TAM includes essential elements that enhance the objectives of this study. Its predictive power comes from its ability to link two important dimensions: the psychological, which includes behavioral goals, and the technical, which includes judgements of usefulness and simplicity of use [62]. Understanding the adoption of AI ChatGPT in metacognitive self-regulated learning contexts requires an important understanding of these relationships. Furthermore, TAM provides the flexibility to adjust and change its elements to fit particular contexts and technologies [63]. The model must be flexible to accommodate the special characteristics and needs of incorporating AI into metacognitive self-regulated learning contexts. To accurately capture the primary factors influencing ChatGPT adoption in the context of metacognitive self-regulated learning, it is imperative to extend the existing technology acceptance and adoption models, such as TAM, as AI applications like ChatGPT in this domain may present distinct characteristics compared to other information technology contexts. taking these factors into account. It is not very popular in the educational field, especially for pre-service programs in higher education. As a result, behavioral intention is the dependent variable in this model. Students' assessments of the usability and convenience of use of AI technology are critical determinants of their behavioral intention, as shown in Fig. 1. Furthermore, behavioral purpose is modelled as directly preceding metacognitive self-regulated learning. External factors that affect how easy and helpful people perceive ChatGPT are also included in the research model. These factors include personal competence [62,63], social influence [61], perceived AI trust [64], perceived usefulness [44,59], perceived enjoyment [51,58], perceived AI intelligence and attitude towards using ChatGPT [44,51,58]. Many important aspects affect students' behavioral intention to utilize ChatGPT in the educational context [65], which influences the adoption of AI for metacognitive self-regulated learning [63,66]. Fundamentally, metacognitive self-regulated learning is the capacity of students to autonomously oversee and assess their learning, which is critical for successfully integrating artificial intelligence (AI) support into their daily learning regimens [64,65]. A key component of the Technology Acceptance Model (TAM) is behavioral intention, which emphasizes how important it is for students to be willing and determined to use ChatGPT for their metacognitive self-regulated learning [44,67]. Personal competence, reflecting students' self-perceived skills in using AI for learning, assumes significance in this context, as does the influence of peers and social networks, encapsulated by the social influence factor [51,58,68]. Additionally, perceived trust in AI systems and perceived AI usefulness are pivotal as they address students' confidence in the technology and their perception of its value in enhancing their metacognitive self-regulated learning [64]. Moreover, the perceived enjoyment of using ChatGPT, their perception of AI intelligence, and their overall attitude toward AI integration collectively shape the adoption landscape. ChatGPT contributes to improved metacognitive self-regulation in learners by providing a range of features that aid in regulating their thinking, motivation, and actions [[69], [70]]. Moreover, the flexibility and convenience offered by computer-assisted and mobile-assisted learning can enhance students' enjoyment and self-efficacy or personal competency, metacognition and self-learning skills ultimately resulting in increased participation in the learning process [71]. Yilmaz and Karaoglan Yilmaz [72] has proposed that AI-powered tools and environments can enhance metacognition and self-learning power by interacting with students, offering personalized support and feedback as they learn programming. In the Chinese context, an et al. [[73], [74]] discovered that technology acceptance significantly impacted middle school students' self-regulated learning (SRL), with information management. These factors are adopted in the framework to comprehensively elucidate the multifaceted nature of AI adoption for metacognitive self-regulated learning in the educational setting. The subsequent sections delve into the formulation of the hypotheses that underpin this model.

### 2.1 Description of constructs and hypotheses development

**Fig. 2:**
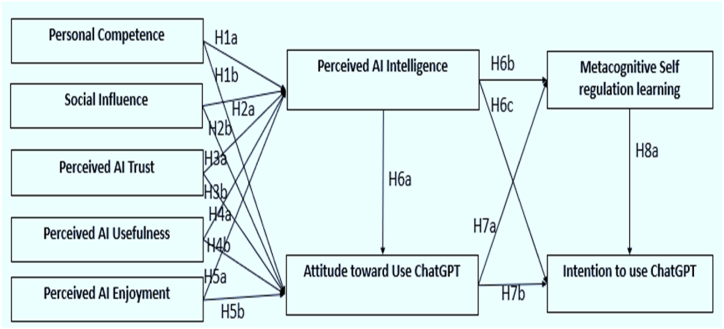
Constructs and their paths.

Numerous factors affect how AI technologies are used in learning contexts, especially the ChatGPT tool for future teachers. These factors are set according to the nature of our study so that factors are easily understandable for researchers. They consist of the following: perceived AI trust, perceived AI enjoyment, perceived AI usefulness and social influence, Personal competency, Perceived AI intelligence, Metacognitive self-regulated learning, attitude toward the use of ChatGPT and finally, behavior intention to use the ChatGPT. Together, these factors impact individuals’ perceptions of using the AI tool ChatGPT to improve the MSRL in the teaching and learning process, affecting their willingness to adopt and utilize ChatGPT [59,73]. The theoretical model underpinnings of technology acceptance, the role of metacognition in education, and the specific factors influencing pre-service teachers' adoption of the AI ChatGPT tool are the basis for the empirical investigation conducted in this study. This study looks into how these factors interact in the context of teacher education to integrate AI into teacher training programs better. See Fig. 2. Showing constructs and proposed hypotheses relationships.

#### 2.1.1 Personal competence

In this sense, "personal competence" refers to self-efficacy, which is how someone feels about their knowledge, skills, and talents. It has to do with one's level of self-assurance and competence in relation to one's capacity for metacognitive self-regulation [62,63]. When it comes to AI tools like ChatGPT, a person with high personal competency will probably approach them confidently, seeing them as an addition to their cognitive abilities rather than a replacement. Bandura's [75] research on self-efficacy theory highlights how one's perceptions of one's competence influence their ideas about one's capacity to carry out particular tasks. High levels of personal competency or self-efficacy are linked to increased drive and tenacity in facing difficulties. According to research conducted by Kelly et al. (2023), those who have higher levels of self-efficacy might be more receptive to employing AI tools [64]. The way that people feel about utilizing ChatGPT is also influenced by their competency [64]. People are more likely to view employing AI tools for metacognitive self-regulated learning favorably if they have confidence in their abilities [34,75].

H1a There is a positive relationship between Personal Competence and Perceived AI Intelligence.

H1b Personal Competence positively influences attitude toward using ChatGPT.

#### 2.1.2 Social influence

The impact of outside influences, such as peers, teachers, or society norms, on a person's choice to use AI tools is represented by social influence in the model [45]. It includes other people's influence on a person's beliefs and actions related to embracing technology. People impacted by their social networks or educational settings are more inclined to adopt AI tools if individuals in their immediate vicinity support their use [41,76]. Peers and social networks have a well-documented impact on technological uptake in literature [41,77,[78], [79]]. Research on innovation diffusion theory conducted by Rogers [80] highlights how social systems influence technology adoption. Because people tend to imitate the beliefs and behaviours of those around them, it affects how AI intelligence is perceived. Social influences also influence an individual's attitude about using ChatGPT. ChatGPT can be positively viewed as a tool for metacognitive self-regulated learning if educators or peers encourage the usage of AI tools.

H2a Social Influence is positively associated with Perceived AI Intelligence.

H2b Social Influence positively affects Attitude toward using ChatGPT.

#### 2.1.3 Perceived AI trust

People's perception of how trustworthy, reputable, and dependable AI technologies like ChatGPT are reflected in their perceived level of AI trust [81]. People must feel safe and confident in the technology's skills and moral application, which makes trust a crucial component of technology adoption [82]. AI tool adoption is encouraged by a high degree of trust in these technologies. Trust plays a widely recognized role in the adoption of technology. The significance of trust in e-commerce transactions is investigated in research [82,83]. Research on artificial intelligence (AI) highlights the importance of consumers' faith in technology for it to be adopted [84]. Given the strong correlation between perceived AI capabilities and trust, this concept affects how intelligent AI is viewed. Their perception of AI trust also influences how someone feels about utilizing ChatGPT. People who believe in the AI tool are inclined to be more optimistic and receptive to using it for metacognitive self-regulated learning.

H3a Perceived AI Trust is positively related to Perceived AI Intelligence.

H3b Perceived AI Trust positively influences attitude toward using ChatGPT.

#### 2.1.4 Perceived AI usefulness

The term "perceived AI usefulness" describes a person's assessment of the worth and efficiency of AI capabilities [85]. It includes the idea that metacognitive self-regulation learning can be improved and made more effective and efficient using AI technologies such as ChatGPT. People are more willing to use AI tools if they believe they will enhance their learning process. One of the main ideas of Davis's Technology Acceptance Model (TAM) is perceived effectiveness. Research by Davis et al. and Venkatesh et al. highlights the critical role that perceived usefulness plays in technology adoption [44,45]. Jauk et al. research emphasizes the importance of perceived usefulness in AI adoption in this scenario. It affects how people perceive AI tools because people tend to equate intelligence with usefulness [[85], [86], [87]]. The degree to which someone feels AI is valuable influences how they think about ChatGPT. People are more inclined to feel positive about using the tool when they believe it can improve metacognitive self-regulated learning [4,43,88].

H4a Perceived AI Usefulness is positively linked to Perceived AI Intelligence.

H4b Perceived AI Usefulness positively affects attitude toward using ChatGPT.

#### 2.1.5 Perceived AI enjoyment

People's perceptions of how much AI is entertaining and engaging determine how much they find it fun to use these products [51,58]. Reliability and continuing technology usage are strongly correlated with enjoyment [89]. Over time, people are more likely to stick with ChatGPT if they find the procedure enjoyable for metacognitive self-regulated learning. Teo et al. [90] research explores the significance of reported enjoyment in the context of online learning systems [91]. Research emphasizes the impact of happiness on the adoption of AI tools. Moreover, Venkatesh [92] discovered that the impact of enjoyment on the perception of ease of use becomes more pronounced as users accumulate direct experience with the system over time. These observations suggest that users' perception of ease of use is influenced by their sense of enjoyment while using the system. Davis et al. [49] established that usefulness and enjoyment play vital roles in determining behavioral intention, and Venkatesh [92] demonstrated that enjoyment affects perceived usefulness through ease of use. In this context, perceived usefulness reflects the belief in how technology can enhance productivity and effectiveness. Notably, the perception of enjoyment has been consistently linked to the intention to use computers [24,89,[90], [93]]. Since enjoyment is linked to favorable qualities, it affects how AI intelligence is perceived. Individuals' attitudes toward ChatGPT usage are also influenced by their perceived enjoyment of AI. Positive attitudes and receptiveness to the tool's application for metacognitive self-regulated learning are more prevalent among people who derive pleasure from the process of utilizing it [68,92].

H5a Perceived AI Enjoyment is positively associated with Perceived AI Intelligence.

H5b Perceived AI enjoyment positively influences attitude toward using ChatGPT.

#### 2.1.6 Perceived AI intelligence

The degree to which people think AI tools like ChatGPT are intelligent, capable of solving problems, and cognitive in general is known as perceived AI intelligence [88]. This construct reflects how the user perceives the AI tool's ability to offer perceptive and intelligent assistance for metacognitive self-regulated learning [94]. Perceived intelligence is considered as a component of perceived utility and ease of use in research under the TAM framework. Perceived intelligence is crucial in influencing user attitudes and adoption, according to research on AI and human perception [95]. This model shows that personal skills directly influence the perception of AI intelligence. People who perceive AI tools such as ChatGPT as complimentary rather than threatening are more likely to attribute intelligence to them when they feel competent in their abilities. An individual's perception of AI intelligence directly impacts their attitude towards ChatGPT usage [95]. People are more likely to be amenable to adopting AI tools for metacognitive self-regulated learning when they believe the technology to be smart. Their perception of AI intelligence also influences an individual's metacognitive self-regulation learning. People who believe ChatGPT to be smart might depend on it to improve their metacognitive techniques and self-control. The likelihood that someone will use ChatGPT is directly impacted by how they perceive AI intelligence. People who think highly of the instrument are more likely to plan to utilize it for metacognitive self-regulated learning.

H6a Perceived AI Intelligence positively predicts attitude toward using ChatGPT.

H6b Perceived AI Intelligence positively influences Metacognitive Self-regulation Learning.

H6c Perceived AI Intelligence is positively associated to use ChatGPT.

#### 2.1.7 Attitude toward using ChatGPT

The term "attitude toward using ChatGPT" describes a person's general assessment of and emotional state toward using ChatGPT for metacognitive self-regulated learning. An optimistic outlook implies a good propensity to interact with the instrument. Within Davis's [45] Technology Acceptance Model (TAM), attitude is a key concept. Many studies that use the Technology Acceptance Model (TAM) highlight attitude's critical role in predicting technology acceptance, including those by Ref. [96]. The behavioral psychology research of Fishbein and Ajzen [97] offers a theoretical framework for comprehending how attitude affects behavioral intentions. Learning of metacognitive self-regulation is influenced by an individual's attitude toward using ChatGPT. Positivity may increase one's willingness to use the tool's metacognitive methods. An individual's attitude towards using ChatGPT directly impacts their intention to use the technology. Positivity increases the likelihood of intending to use ChatGPT for metacognitive self-regulated learning.

H7a Attitude toward using ChatGPT positively influences metacognitive self-regulation learning.

H7b Attitude toward using ChatGPT is positively linked to Intention to use ChatGPT.

#### 2.1.8 Metacognitive self-regulation learning

People actively employ self-regulation processes and metacognitive strategies to enhance their learning experiences [73]. Metacognitive self-regulation learning is the term for this [98]. This construct reflects the application of metacognitive knowledge and skills to maximize and manage one's learning [63,66]. The concept of metacognition is well-established in educational psychology [99]. Research by Flavell popularized the term "metacognition" and highlighted its function in learning that is self-regulated [5]. The value of metacognitive self-regulation in educational settings is covered in studies by Refs. [19,22,63,88,95,99]. Furthermore, studies by Lai et al. emphasize how important metacognition is to enhance learning results [35]. Lastly, the intention of an individual to use ChatGPT is influenced by metacognitive self-regulation learning. People are more likely to use the tool in the future for learning experiences if they use it to improve their metacognitive skills and self-regulation [34,100].

H8a Metacognitive Self-regulation Learning is positively associated with Intention to use ChatGPT.

#### 2.1.9 Intention to use ChatGPT

The term "intention to use ChatGPT" describes a person's desire and resolve to utilize ChatGPT for metacognitive self-regulated learning [87]. Based on contemporary attitudes and views, it indicates a user's proactive propensity to keep using the product. One of the main ideas of the Technology Acceptance Model (TAM) is the intention to use technology [49]. Venkatesh et al. and Davis conducted research that emphasizes the significance of intention as a predictor of technology adoption [91,96]. Studies by Na et al. [99] show user intention plays an important role in adopting AI [101]. The acceptability and application of ChatGPT for metacognitive self-regulated learning among pre-service teachers is contingent upon each of the theoretical model's constructs. The framework of the study is well-founded because these constructs are backed by previous research in the domains of technology acceptance, AI adoption, metacognition, and related areas [99].

## 3 Research design

**Fig. 3:**
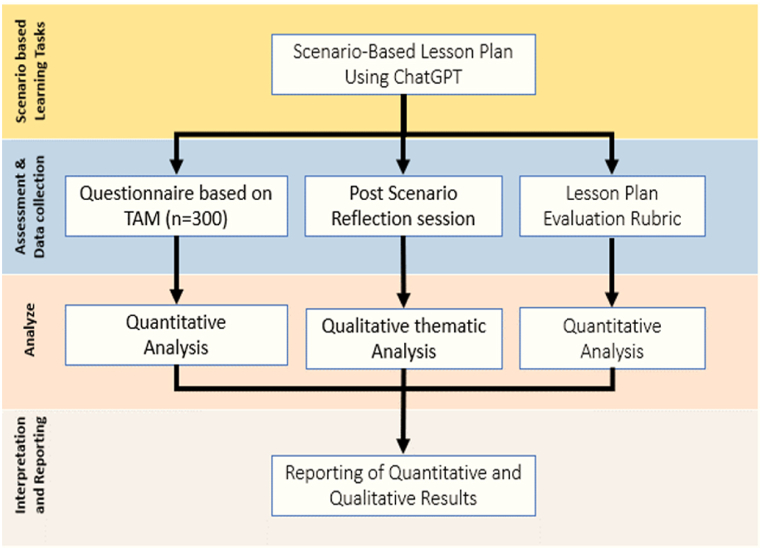
Flowchart of research methodology.

The research methodology adopts a mixed-method approach, encompassing qualitative and quantitative methods, to investigate pre-service teachers' acceptance of ChatGPT for metacognitive self-regulated learning. The study unfolds through a multi-layered process, as illustrated in Fig. 3. Initially, participants engage in a scenario-based learning task where they utilize ChatGPT to create lesson plans, utilizing their metacognitive and self-learning abilities (See scenario-based tasks details, Appendix A and Table 1). Subsequently, the research employs three key components for data collection to gauge acceptance levels. Firstly, participants complete a structured questionnaire designed according to the proposed extended TAM acceptance framework (See complete Questionnaire, Appendix B). We employed a convenience sampling method to select participants from the School of Education department at UTM University. Participants were chosen based on their availability and willingness to participate. All selected pre-service teachers provided informed consent before participating. The population size was determined using the criteria established by Hair et al. (2019), which required five to ten responses for each item.

**Table 1:** Scenario-based learning Tasks (MSRL strategies using ChatGPT).

MSRL Strategy	Scenario No	Scenario Name	Summary
Activation of prior content knowledge	S01	ChatGPT Lesson Recall Assistant	ChatGPT assists pre-service teachers in recalling prior lesson content relevant to their current lesson plan creation.
Setting goals	S02	ChatGPT Learning Objective Setter	ChatGPT helps pre-service teachers set clear learning objectives for their lesson plans and suggests action plans.
Activation of task value and interest	S03	ChatGPT Engagement Enhancer	ChatGPT provides engaging content ideas and methods to keep students interested in the lesson plans being created.
Selection and adaptation of cognitive strategies	S04	ChatGPT Resource Recommender	ChatGPT recommends suitable study resources, materials, and strategies for pre-service teachers' lesson plans based on their individual needs and preferences.
Metacognitive monitoring	S05	ChatGPT Progress Tracker	ChatGPT monitors pre-service teachers' progress in creating lesson plans, providing feedback and suggestions for improvement.
Selection strategies for managing motivation and affect	S06	ChatGPT Motivation Booster	ChatGPT offers motivational content and insights to keep pre-service teachers motivated and enthusiastic while creating lesson plans.
Help-seeking behavior	S07	ChatGPT Help Desk Assistant	ChatGPT answers questions and provides guidance to pre-service teachers in real-time as they work on their lesson plans.
Reviewing	S08	ChatGPT Review and Feedback	ChatGPT allows pre-service teachers to review and revise their lesson plans, offering feedback and recommendations based on best practices.
Self-evaluation	S09	ChatGPT Self-Assessment Aid	ChatGPT generates self-assessment quizzes for pre-service teachers to evaluate the quality and effectiveness of their lesson plans.
Self-satisfaction	S10	ChatGPT Self-Recognition	ChatGPT helps pre-service teachers recognize their achievements and improvements in lesson plan creation, reinforcing their self-confidence

Given the 41 items in the survey, a sample size ranging from 164 to 205 participants was considered adequate [102]. Of the 320 returned questionnaires, 300 respondents, constituting 93.8 % of the total respondents, reported having experience with ChatGPT [102]. Secondly, post-task reflection sessions allow participants to share their experiences with ChatGPT for metacognitive self-regulated learning (See Reflective Questions, Appendix C). In this step, we selected interviews as the important data source for their richness in analysis [103]. The semi-structured format ensured flexibility, adaptability, and participant comfort [[103], [104], [105]]. This method facilitated direct communication with ChatGPT participants, providing unique understandings [[106], [107]] and allowing in-depth exploration of complex phenomena [108]. Real-time question adjustments and rapport building were facilitated [109]. Despite advantages, interviews have limitations such as time consumption and potential biases [108]. However, we adopt the convenience sampling methods for participant recruitment [110]. The third component involves evaluating the quality of the generated lesson plans using predefined rubric criteria to assess practical application (See Rubric, Appendix D). Data triangulation is implemented to gather insights from lesson plans, surveys, and reflection sessions, facilitating a comprehensive understanding of pre-service teachers' acceptance. After data collection, analysis is performed, leading to conclusions.

### 3.1 Scenario-based learning task development

To craft AI scenarios focusing on learning task creation, our team, comprised of three educational technology experts and one AI specialist with a collective research experience of 12.5 years [76,92], employed a collaborative "brainwriting" approach. Using a theoretical framework for self-regulated learning (SRL), they iteratively developed AI scenarios on Google Docs, ensuring alignment with metacognitive SRL techniques. This process continued until a consensus was reached, followed by a focus group interview with four educational professionals. The experts assessed each scenario's potential to enhance students' metacognitive SRL in learning task and lesson plan creation through video conferencing. Their insights guided scenario refinement, emphasizing the higher education context [1]. This collaborative effort yielded tailored AI scenarios, detailed in Table 1, designed to impact students' metacognitive SRL in higher education positively. Further the assessment of metacognition and self-regulated learning, drawing from Dinsmore and colleagues' research overview, used Self-report measures are widely used for self-regulated learning (73 %), while metacognition assessment is more diverse, with less reliance on self-report measures (24 %). Structured interviews were utilized to provide a context-specific approach, addressing the limitations of surveys [1,10]. This approach allows a deeper understanding of context-specific strategies. Additionally, we assessed the practical application of these constructs in real-world educational scenarios, adding depth to our research. Our methodology ensures robust findings and enriches our understanding of metacognition and self-regulated learning.

### 3.2 Data collection, analysis process and ethics

#### 3.2.1 Structured survey questionnaire

The questionnaire employed in this study consisted of two main sections. The first section gathered demographic information encompassing gender, age, and educational level. In the second section, the structured questionnaire used in this study was carefully designed based on the proposed acceptance framework. This survey instrument collected quantitative data on participants' acceptance of ChatGPT for metacognitive self-regulated learning. To develop the questionnaire, existing constructs from the Technology Acceptance Model (TAM) were integrated into the research framework containing 41 items thoughtfully selected from prior research to ensure content validity. The study investigates various constructs related to individuals' perceptions and attitudes toward AI, particularly focusing on ChatGPT, and outlines the number of items and sources of adoption for each. Personal Competence, assessing perceived competency in AI usage, comprises five items from sources [59,62]. Social Influence, evaluating the impact of external factors, includes five items sourced from Refs. [[110], [111], [112]]. Perceived AI Trust, measuring trust in AI technologies, incorporates five items based on source [64]. Perceived AI Usefulness, assessing perceptions of AI utility, consists of five items from Ref. [44], and [61]. Perceived AI Enjoyment, measuring subjective experiences with AI, comprises five items from Refs. [51,58,113]. Perceived AI Intelligence, evaluating perceptions of AI cognitive capabilities, is based on four items from Ref. [84], and [90]. Attitude toward using ChatGPT, gauging overall attitudes toward ChatGPT, includes four items sourced from Refs. [110,111]. Metacognitive Self-regulation learning, assessing metacognitive strategies for self-regulated learning, consists of four items from Refs. [62,101]. Intention to use ChatGPT, exploring individuals' intentions to use ChatGPT, includes four items adopted from Refs. [102,103].

Ensuring that it encompasses factors relevant to AI adoption in education. The questions in the questionnaire were carefully selected from prior research to ensure content validity. To assess the reliability of the questionnaire, a pilot test was administered to 35 pre-service students, and the Cronbach's alpha method, with a recommended cutoff point of 0.7, was employed to evaluate its internal consistency [114,115]. The final Cronbach's alpha values for each construct exceeded the threshold, affirming the data's reliability for further analysis. We analyzed the data using SPSS, which allowed us to examine descriptive and inferential statistics, providing valuable participant insights.

We used Smart PLS 4.0 for the structural modelling phase of the analysis. This was done per the well-established two-step approach suggested by other research [116,115,[117], [118]]. In the first stage of the research study, the researchers focused on developing, converging, and evaluating the discriminant validity of the measurements. After that, in the second step of the research process, an in-depth examination of the model's structure was carried out.

#### 3.2.2 Post-task reflection interview

The post-task reflection sessions were an integral component of data collection, allowing participants to provide qualitative insights and share their experiences regarding using ChatGPT for metacognitive self-regulated learning [86].Drawing from past works, five open-ended questions were crafted to explore various dimensions [119], including ChatGPT's merits, cons, and user's ChatGPT experiences. Interviews were guided by research team open-ended questions. Thirty senior participants responded in writing on reflective diaries. Participants averaged 2 h 30 min (SD = 5 min) for interviews. Spending 2 min on lesson ideas and interview responses examined the subject holistically. Qualitative narratives illuminated participants' viewpoints. The cleaned data underwent thematic analysis by Braun & Clarke [120], ensuring a comprehensive understanding of factors influencing ChatGPT use. This approach facilitated identifying and interpreting themes in the qualitative data, providing insights into participants' beliefs and thoughts on ChatGPT. These interviews were transcribed and analyzed [121]. A systematic process includes data familiarization, coding, topic development, and interpretation. In the interviews, we clarified ChatGPT's capabilities and relevance to MSRL to ensure participants understood the AI applications [1,85].

#### 3.2.3 Lesson plan evaluation checklist

The third stage of the data-gathering process was evaluating the quality of the lesson plans that the participants had prepared using a rubric that had been predefined with certain criteria designed [122]. In order to construct this checklist-based tool, a set of criteria was first defined. Next, we analyzed the 30 lesson plans that included 10 % of the whole sample, taking into consideration the desirable characteristics of a well-structured lesson plan that represents effective metacognitive self-regulated learning. To assess lesson plan quality and identify success themes, rubric scores were quantitatively analyzed [122]. To understand participants' acceptance, experiences, and practical usage of ChatGPT in metacognitive self-regulated learning, data triangulation was used.

#### 3.2.4 Research ethics

These three data collection tools, employed in a mixed-method approach, enabled a thorough exploration of the research questions by combining quantitative and qualitative data, ultimately contributing to a holistic analysis of the acceptance and utilization of ChatGPT in metacognitive self-regulated learning. Ethical considerations have significance in research. Researchers are morally and legally bound to follow these considerations. Consequently, in this investigation, the researchers sought prior approval from the participants. The subjects were offered free will to participate in the study or not. Similarly, the researchers maintained the confidentiality and anonymity of participants’ views.

## 4 Results and analysis

### 4.1 Demographics data analysis

The study of three hundred participants (N = 300) information consisted of individuals aged between 20 and 30 years, encompassing the entire study population. In terms of gender distribution, the majority of participants identified as male (n = 275, 91.67 %), while a smaller percentage identified as female (n = 30, 8.33 %). Furthermore, a substantial portion of the participants reported prior experience with AI in teaching and learning contexts, with 66.67 % (n = 200) confirming their usage. Additionally, 60 % of the participants (n = 180) demonstrated knowledge of ChatGPT, a specific AI tool, while 40 % (n = 120) indicated their lack of awareness regarding ChatGPT. These demographic findings provide insights into the composition of the study sample, highlighting the age range, gender distribution, prior AI experience, and ChatGPT familiarity among participants.

### 4.2 Assessment of the research model

#### 4.2.1 Descriptive analysis (mean, SD)

The analysis of mean and standard deviation (SD) for the questionnaire constructs revealed generally favorable perceptions among the participants regarding personal competence, social influence, perceived AI trust, AI usefulness, AI enjoyment, AI intelligence, attitude toward using ChatGPT, metacognitive self-regulation learning, and intention to use ChatGPT (see Table 2 for Mean and SD). Specifically, participants exhibited high levels of personal competence, trust, and perceived usefulness of AI, as indicated by mean scores above 4.0. The highest preference, with a mean score of 4.1, was observed for "Attitude Toward Use ChatGPT." While the overall trends were positive, the moderate variability in SD values suggested diverse responses and attitudes within the participant group. These findings underscore the generally positive disposition of participants toward ChatGPT and metacognitive self-regulated learning, offering valuable insights for further investigation.

**Table 2:** Mean, Standard Deviation, and Factor loading results.

Constructs	Item	M	SD	Factor loading
**PC**	PC1	4.12	1.14	0.77
	PC2	4.06	0.98	0.82
	PC3	4.09	0.94	0.80
	PC4	3.94	0.94	0.73
	PC5	3.96	1.05	0.67
**SI**	SI1	3.99	1.01	0.84
	SI2	3.95	1.02	0.85
	SI3	4.01	0.97	0.84
	SI4	3.93	0.96	0.82
	SI5	3.92	0.96	0.79
**PT**	PT1	3.85	1.04	0.78
	PT2	4.16	0.91	0.79
	PT3	4.05	01.0	0.87
	PT4	4.2	0.87	0.83
	PT5	4.23	0.91	0.77
**PU**	PU1	4.03	0.95	0.71
	PU2	4.03	01.0	0.83
	PU3	4.11	1.01	0.80
	PU4	3.89	1.07	0.76
	PU5	04.0	0.92	0.77
**PE**	PE1	4.05	0.89	0.78
	PE2	4.07	0.90	0.79
	PE3	3.94	1.05	0.81
	PE4	4.03	1.02	0.83
	PE5	3.97	1.03	0.81
**PAI**	PAI1	4.05	0.97	0.83
	PAI2	04.1	0.96	0.85
	PAI3	4.05	0.97	0.85
	PAI4	4.16	0.9	0.84
**ATU**	ATU1	04.1	1.05	0.81
	ATU2	4.05	1.0	0.83
	ATU3	4.19	0.94	0.84
	ATU4	3.95	01.0	0.72
**MSR**	MSR1	4.04	1.01	0.84
	MSR2	4.06	0.99	0.87
	MSR3	4.11	0.98	0.83
	MSR4	4.09	0.97	0.85
**BIU**	BIU1	4.07	1.01	0.87
	BIU2	4.11	01.0	0.85
	BIU3	4.17	0.96	0.88
	BIU4	4.19	01.0	0.85

#### 4.2.2 Reliability and convergent validity analysis

In line with established guidelines for evaluating convergent validity, our study carefully examined factor loadings, average variance extracted (AVE), and composite reliability (CR) [68,116,122]. According to these guidelines [116,122]., factor loadings are expected to exceed 0.70, while CR should surpass 0.7, and AVE should be higher than 0.5 [116,122]. Our structural equation modeling (SEM) analysis, as outlined in Table 3, provides crucial insights into the outcomes of our research model, in accordance with these guidelines. Notably, the constructs consistently demonstrated robust internal consistency and reliability, supported by Cronbach's alpha values ranging from 0.81 to 0.89. These values surpass the recommended threshold of 0.7, affirming strong internal consistency [102,123]. Additionally, our constructs exhibited commendable convergent validity, with AVE values ranging from 0.82 to 0.89, indicating effective accounting of variance in each construct [116,122]. Our factor loadings, representing the strength of the relationship between items and their respective constructs, consistently exceeded the recommended threshold of 0.70, underscoring the significance of these items in measuring their respective constructs [116,122]. These robust findings collectively underscore the reliability and validity of our measurement model and highlight the appropriateness of these constructs and their items within our research framework. The construct reliability, AVE, and CR are presented in Table 3, and factor loading values, as presented in Table 2, indicate the rigorous evaluation of the measurement model's reliability and validity in our study [61,77,124].

**Table 3:** Convergent validity results (Cronbach's alpha, AVE, and CR).

Constructs	Reliability	AVE	CR
Personal Competence	0.81	0.82	0.64
Social Influence	0.88	0.88	0.74
Perceived AI Trust	0.87	0.87	0.72
Perceived AI Usefulness	0.86	0.86	0.71
Perceived AI Enjoyment	0.81	0.82	0.58
Perceived AI Intelligence	0.86	0.87	0.65
Attitude toward use ChatGPT	0.87	0.87	0.65
Metacognitive Self-regulation learning	0.83	0.83	0.6
Intention to use ChatGPT	0.89	0.89	0.69

Table 4 presents an analysis of the R-squared values, shedding light on the relationships between exogenous and endogenous constructs within our study [102,117]. In the context of our investigation into "Intention to use ChatGPT (IU)," the R-squared value of 0.72 indicates that a significant 72 % of the variance in pre-service teachers' intention to use ChatGPT for metacognitive self-regulated learning is attributable to the exogenous variables considered, underscoring their substantial impact. Similarly, "Behavioral Intention to Use ChatGPT (BIU)" showcases an R-squared value of 0.61, emphasizing that 61 % of the variance in pre-service teachers' intentions to employ ChatGPT for metacognitive self-regulated learning is elucidated by these selected exogenous variables. Furthermore, the construct "Metacognitive Self-Regulation Learning (MSR)" boasts an R-squared value of 0.69, signifying that the chosen exogenous variables can explain 69 % of the variance in pre-service teachers' metacognitive self-regulation learning. Lastly, "Perceived AI Intelligence (PAI)" reveals an R-squared value of 0.64, highlighting that these variables clarify 64 % of the variance in pre-service teachers' perceptions of ChatGPT's intelligence. These results collectively underscore the considerable influence of the exogenous factors on pre-service teachers' intentions, attitudes, and perceptions concerning ChatGPT for metacognitive self-regulated learning, emphasizing the multi-faceted nature of technology acceptance in the educational context and its implications for educational practices and research.

**Table 4:** Model fitness score -**R2** of the endogenous latent variables.

Variables	R-square	R-square adjusted
Attitude toward Use (ATU)	0.72	0.71
Intention to use ChatGPT (BIU)	0.61	0.61
Metacognitive self-regulated learning (MSR)	0.69	0.69
Perceived AI intelligence (PAI)	0.64	0.64

#### 4.2.3 Discriminant validity analysis

Ensuring discriminant validity is a crucial aspect of our study, designed to distinguish between various sets of definitions and their associated measurements, thus confirming that the constructs effectively represent distinct latent concepts without significant overlap. As anticipated in our research [125,126], the results of our measurement model show strong discriminant validity, surpassing the recommended threshold of 0.50 and achieving statistical significance at p = 0.001. We employed two widely recognized criteria, the Heterotrait Monotrait Ratio (HTMT) and the Fornell-Larcker criterion [125,126], to rigorously evaluate discriminant validity. Specifically, the HTMT ratio method, presented in Table 5, reaffirms discriminant validity. All off-diagonal values are comfortably below the threshold of 0.85, which provides compelling evidence for the clear distinction between the constructs incorporated into our measurement model. This confirmation ensures that the variables we studied effectively measure separate latent concepts and are suitable for use in this research.

**Table 5:** Discriminant Validity (HTMT ratio).

	ATU	BIU	MSR	PAI	PC	PE	PT	PU	SI
**ATU**									
**BIU**	0.87								
**MSR**	0.85	0.84							
**PAI**	0.89	0.76	0.87						
**PC**	0.83	0.72	0.74	0.69					
**PE**	0.83	0.7	0.8	0.8	0.72				
**PT**	0.89	0.75	0.83	0.83	0.78	0.79			
**PU**	0.82	0.69	0.82	0.76	0.82	0.76	0.77		
**SI**	0.87	0.76	0.87	0.81	0.73	0.76	0.78	0.75	

In accordance with the Fornell-Larcker criterion values presented in Table 6, it is crucial to highlight that the diagonal elements represent the square root of the Average Variance Extracted (AVE) for each construct. A noteworthy observation arises when comparing these diagonal values with the off-diagonal values, which indicate correlations between constructs. In all cases, the diagonal values consistently exceed the off-diagonal values for each pair of constructs [125]. This compelling observation firmly confirms the discriminant validity of the constructs, as each construct's AVE significantly surpasses its squared correlation with other constructs. The HTMT ratio and the Fornell-Larcker criterion methods provide robust evidence reinforcing the discriminant validity of the constructs within our study. These collective findings affirm that these constructs effectively measure distinct latent variables, ensuring the absence of multicollinearity or overlapping influences [[125], [126], [127]].

**Table 6:**
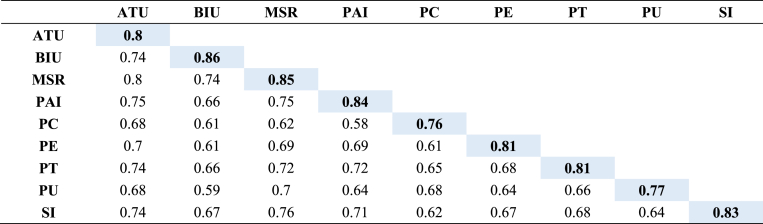
Discriminant Validity (Furnell larker Criterion).

#### 4.2.4 Hypotheses testing (path analysis of the structural model)

**Fig. 4:**
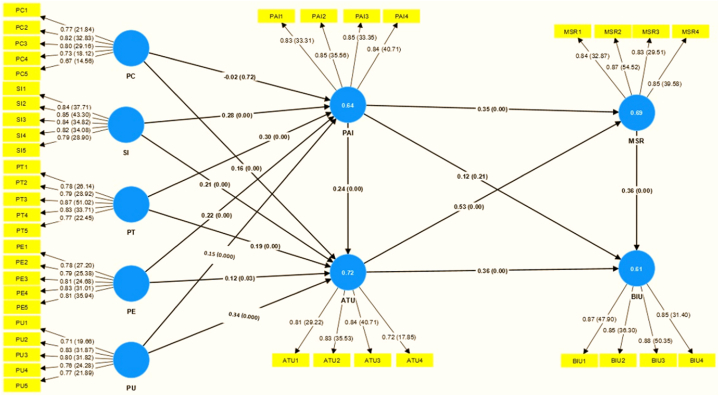
Structural model (path coefficients).

The structural equation model analysis was conducted using Smart PLS, applying the maximum likelihood estimation approach to explore the interrelationships among various theoretical constructs within the structural model [67,70,73]. The study tested multiple hypotheses to understand the complex interplay of factors influencing metacognitive self-regulated learning and the acceptance of AI technology for educational purposes. The findings, as presented in Table 7, indicate that the model exhibited a moderate level of predictive capability, accounting for approximately 63 % of the variance in technology acceptance rates (See Fig. 4 showing complete results of the hypothesis).

**Table 7:** Hypothesis testing (Path, T-Value, and *P*-value).

Relationships	Original sample	T statistics	P values	Decision
H1a = PC - > PAI	−0.020	0.360	0.720	Rejected
H1b = PC - > ATU	0.160	3.330	0.000	Supported
H2a = SI - > PAI	0.280	3.950	0.000	Supported
H2b = SI- > ATU	0.210	3.480	0.000	Supported
H3a = PT - > PAI	0.300	3.780	0.000	Supported
H3b = PT - > ATU	0.120	2.170	0.030	Supported
H4a = PU - > PAI	0.150	3.040	0.000	Supported
H4b = PU - > ATU	0.340	3.980	0.000	Supported
H5a = PE - > PAI	0.220	3.380	0.000	Supported
H5b = PE - > ATU	0.120	2.170	0.030	Supported
H6a = PAI - > ATU	0.240	3.780	0.000	Supported
H6b = PAI - > MSR	0.350	5.270	0.000	Supported
H6c = PAI - > BIU	0.120	1.240	0.210	Rejected
H7a = ATU - > MSR	0.530	8.600	0.000	Supported
H7b = ATU - > BIU	0.360	4.040	0.000	Supported
H8a = MSR - > BIU	0.360	3.450	0.000	Supported

The results of the hypothesis testing revealed significant insights into the relationships between various constructs within our study. Several hypotheses received strong support, including H1b, H2a, H2b, H3a, H3b, H4a, H4b, H5a, H5b, H6a, H6b, and H7a, indicating their significance in shaping metacognitive self-regulated learning and AI technology acceptance. In these instances, metacognitive self-regulated learning (MSR) and the acceptance of AI technology (ATU) were found to be significantly impacted by constructs such as Personal Competence (PC), Social Influence (SI), Perceived AI Trust (PT), Perceived AI Usefulness, Perceived AI Enjoyment (PE), and Perceived AI Intelligence (PAI). <b>H1b:</b> Personal Competence (PC) positively affects AI Technology Acceptance (ATU) (T = 3.330, p = 0.000), supporting this hypothesis. This suggests that educators' self-perception of competence strongly influences their acceptance of AI technology for teaching.<b>H2a:</b> Social Influence (SI) positively affects Perceived AI Intelligence (PAI) (T = 3.950, p = 0.000), supporting this hypothesis. It shows that peers and colleagues significantly impact instructors' assessments of AI's educational intelligence. <b>H2b:</b> Social Influence (SI) positively influences the adoption of AI Technology (ATU) (T = 3.480, p = 0.000), emphasizing social influence's significance in educators' AI technology adoption. This finding strongly supports the concept. H3a and H3b show that Perceived AI Trust (PT) positively affects Perceived AI Intelligence (PAI) (T = 3.380, p = 0.000) and Acceptance of AI Technology (ATU) (T = 2.170, p = 0.030), supporting both hypotheses. These hypotheses illuminate how educators' trust in AI affects metacognitive self-regulated learning and their intention to use AI in education. Trust affects instructors' technology uptake and engagement. <b>H4a:</b> Perceived AI Usefulness (PU) positively increases perceptions of AI intelligence (PAI) (T = 2.170, p = 0.000), highlighting educators' influence on AI intelligence perceptions. Support is high for this hypothesis. <b>H4b:</b> Educators' perceived AI usefulness (PU) favorably affects their attitude to use AI technology (ATU) (T = 3.980, p = 0.000). Although significant, this support is weaker than some other theories. <b>H5a:</b> Educators' enjoyment of AI favorably increases their views of AI's intelligence (PAI) (T = 3.380, p = 0.000). Support is high for this hypothesis. <b>H5b:</b> Educators' delight of utilizing AI favorably increases their acceptance of AI technology for educational applications (T = 2.170, p = 0.030). This hypothesis has strong support, yet it is weaker than others. <b>H6a:</b> Educators' evaluations of AI's intelligence positively affect their adoption of AI technology (ATU) (T = 3.780, p = 0.000). Support is high for this hypothesis. <b>H6b:</b> Educators' assessments of AI's intelligence positively influence metacognitive self-regulated learning (MSR) (T = 5.270, p = 0.000). Support is high for this hypothesis. <b>H6c:</b> Perceived AI Intelligence (PAI) positively correlates Behavioral Intention to Use AI (BIU) (T = 1.240, p = 0.210), demonstrating that educators' opinions of AI's intelligence do not significantly affect their intention to use AI for education. This hypothesis is weak. <b>H7a:</b> Acceptance of AI Technology (ATU) improves Metacognitive Self-Regulated Learning (MSR) (T = 8.600, p = 0.000), supporting this hypothesis. This highlights the importance of educators' acceptance of AI technology in metacognitive self-regulated learning. <b>H7b:</b> Acceptance of AI Technology (ATU) increases Behavioral Intention to Use AI (BIU) (T = 4.040, p = 0.000), supporting this hypothesis. This shows that educators' acceptance of AI increases their intention to employ it for education. <b>H8a:</b> Metacognitive Self-Regulated Learning (MSR) increases Behavioral Intention to Use AI (BIU) (T = 3.450, p = 0.000), supporting this hypothesis.

### 4.3 Post-reflection session (interview findings)

The post-reflection themes from 30 participants reveal a predominantly positive perception of Metacognitive Self-Regulated Learning (MSRL) support across different teaching phases as seen in Table 8. In the Planning phase, a significant majority (83.3%–93.3 %) found ChatGPT helpful for planning lesson content, setting clear objectives, outlining strategies, organizing lesson structure, and offering teaching resources. However, a small percentage (6.7%–10.0 %) reported no noticeable impact on lesson planning and content understanding.

**Table 8:** Summary of Pre services reflections.

Phases	Learners' Perceptions of MSRL Support	No. of Opinions	%
Planning	Helpful for planning lesson content	25	83.3
	Useful for setting clear objectives	26	86.7
	Supportive of outlining strategies	24	80.0
	No noticeable impact on lesson planning	2	6.7
	Beneficial for organizing lesson structure	27	90.0
	No significant influence on content understanding	3	10.0
	Effective in offering teaching resources	28	93.3
Presentation	Supportive of improved lesson clarity	29	96.7
	Beneficial for enhancing content quality	28	93.3
	No substantial effect on presentation skills	2	6.7
	Effective in aligning with learning objectives	26	86.7
	Facilitates sharing and collaboration	15	50.0
	Encourages seeking peer feedback	1	3.3
	Effective in organizing teaching resources	29	96.7
Reflection	Supportive of post-lesson review	28	93.3
	Beneficial for assessing learning outcomes	27	90.0
	Facilitates tracking progress and goals	12	40.0
	Encourages self-assessment and self-awareness	25	83.3
	Boosts motivation for teaching	26	86.7
	Fosters a positive attitude towards teaching	27	90.0

During the Presentation phase, participants overwhelmingly recognized the supportive role of ChatGPT in improving lesson clarity (96.7 %), enhancing content quality (93.3 %), and aligning with learning objectives (86.7 %). It also effectively organized teaching resources (96.7 %). While there was positive feedback on the facilitation of sharing and collaboration (50.0 %) and encouragement for seeking peer feedback (3.3 %), a small proportion (6.7 %) reported no substantial effect on presentation skills.

In the Reflection phase, the majority perceived ChatGPT as supportive for post-lesson review (93.3 %), beneficial for assessing learning outcomes (90.0 %) and encouraging self-assessment, self-awareness, motivation for teaching, and fostering a positive attitude towards teaching (83.3%–90.0 %). However, a moderate proportion (40.0 %) reported that it facilitated tracking progress and goals. These responses indicate a strong positive perception of ChatGPT's contribution to various aspects of teaching, particularly in planning, presenting, and reflecting on lessons. The small percentages reporting no substantial impact or noticeable influence suggest a generally favorable outlook among participants. The reflection tool analysis highlighted positive feedback, identified challenges in interaction that emphasized the need for improved user guidance, and revealed increased confidence among participants in tackling complex topics through ChatGPT. These insights offer valuable considerations for future improvements and refinements.

The evaluation of interview results resonates with the Technology Acceptance framework, revealing a significant acceptance and perceived value of ChatGPT in the field of lesson planning. Participants showcased a positive attitude towards the tool, acknowledging its effectiveness in enhancing various facets of Metacognitive Self-Regulated Learning (MSRL). The identified benefits, such as facilitating lesson planning and improving content quality, align with the core constructs of TAM. This alignment underscores the potential successful integration of ChatGPT into the teaching and learning environment, particularly among pre-service teachers. Their favorable reception of AI.

### 4.4 Evaluation of lesson plans results

The analysis of the lesson plan evaluations in our study provides valuable perceptions into the acceptance of ChatGPT for metacognitive self-regulated learning (MSRL). The results indicate that the majority of the assessed lesson plans exhibited clarity in defining learning objectives, logical content coherence, alignment with intended learning outcomes, and the inclusion of engaging strategies. The finding results demonstrated that 86.7 % of the lesson plans were clear in outlining their objectives, underscoring the effectiveness of ChatGPT in assisting pre-service teachers in effectively planning their lessons to enhance student engagement and meet educational goals. Moreover, a significant percentage of lesson plans (93.3 %) were coherent in their subject matter knowledge and well-aligned with the specified learning outcomes (93.3 %). These findings not only highlight the potential of ChatGPT but also suggest a positive impact on teachers' ability to develop quality lesson plans. However, it's essential to note that there is room for improvement in some instances, particularly in ensuring consistent alignment with learning outcomes and including engaging elements (16.7 % fell short in this aspect). This analysis reinforces the significance of educators' readiness and effective utilization of AI tools like ChatGPT to optimize lesson planning and student engagement, ultimately supporting the principles of metacognitive self-regulated learning.

## 5 Discussion

Our study examines the extended Technology Acceptance Model (TAM) framework to explore educators' understanding of AI-powered ChatGPT and its impact on Metacognitive Self-Regulated Learning (MSRL) in education. The comprehensive mixed-methods approach, integrating survey data, post-reflection themes, and lesson plan evaluations, offers a holistic understanding of AI adoption in educational contexts. The impact of ChatGPT on educators' attitudes, perceptions, and practices in Metacognitive Self-Regulated Learning (MSRL) is complex and varied. In our discussion, we explore the diverse influences uncovered through hypothesis testing, insights from participant reflections, and the analysis of lesson plan evaluations. This thorough examination enables us to understand the multifaceted role of ChatGPT in shaping educational practices, revealing both positive aspects and areas for improvement in supporting metacognitive skills and self-regulation among teachers.

The ChatGPT Adoption section talks about how much and in what ways ChatGPT is used. It tells us about the results of using ChatGPT and discusses overcoming challenges and the good and not-so-good parts of using the tool. It also looks at different factors that affect how much people use ChatGPT, like how confident they feel, what their peers think, and how much they enjoy using it. Understanding these aspects is important to grasp ChatGPT's role and impact in higher education [49].

### 5.1 Personalized competence

The survey results strongly support this theory, showing that educators' self-perceptions of competence affect their acceptance of AI technology. Previous studies supported personal competency on ChatGPT acceptance [62,63]. Our interview findings support technology acceptance theories since educators stressed the necessity of “feeling competent and capable with AI technology”. The lesson plan evaluations also showed how educators with higher “perceived competency used AI technology in their lessons”.

### 5.2 Social influence (SI)

The survey supported this hypothesis, showing that social influence shapes educators' AI intelligence assessments. Social influence on technology uptake is supported by previous research [41,77,78]. Our interviews show colleagues' opinions and experiences shape educators' AI technology perceptions. “Some participants mentioned that they had initially considered using ChatGPT based on recommendations from their peers. This indicated that social influence played a role in their decision to explore AI-driven learning tools”. In lesson plan assessments, educators who respected their colleagues' insights used AI technology more effectively. Our survey supported this hypothesis, emphasizing social influence in educators' AI technology acceptance. Many educators interviewed said they used AI technology because of positive peer experiences.” Participants who collaborated on tasks involving ChatGPT emphasized the social aspect of learning. They shared insights and strategies with peers, highlighting the collaborative nature of using AI tools for metacognitive self-regulated learning”. In lesson plan evaluations, peer-influenced instructors showed stronger aspirations to use AI in their teaching.

### 5.3 Perceived AI trust

Perceived AI trust (PT) is a key aspect in educators' AI technology interactions; thus significantly influencing Perceived AI intelligence and Attitude toward ChatGPT usage, our findings imply that educators' faith in AI considerably affects their perceptions, acceptance, and intentions towards AI technology. Trust is crucial to technology adoption, as shown in the literature and aligned with studies [80,81]. Trust was an essential factor mentioned by participants. They indicated that they trusted the information and responses provided by ChatGPT, which is essential for successful learning. They anticipated long-term benefits from AI-driven support [82,83].

### 5.4 Perceived usefulness

Perceived AI Usefulness (PU) and Intelligence (PAI): The survey data strongly supported this hypothesis, showing that educators' judgements of AI's usefulness strongly influence their perceptions of its intelligence, and it is aligned with other studies [[85], [86], [87]]. In interviews, educators said that seeing AI's practical applications improved their opinion of its intelligence. A recurring theme was the perceived usefulness of ChatGPT in enhancing learning support. Participants reported that “ChatGPT's AI-driven responses provided valuable insights and explanations that enriched their understanding of complex subjects”. As instructors used AI tools to meet learning objectives, lesson plan assessments showed a substantial correlation between usefulness and perceived intelligence. Perceived AI Usefulness (PU) and Attitude AI Use (ATU): Survey results supported this hypothesis significantly less. The interviews showed that educators valued AI when it made teaching and administration easier. “Participants acknowledged the practicality of “ChatGPT in assisting them with lesson planning”. They found the AI's ability to generate content and outline lesson structures as a helpful resource for educators”. This increased usefulness was linked to acceptability, as seen in lesson plan evaluations. Practically using AI in education increased acceptance of AI technology.

### 5.5 Perceived AI enjoyment

Perceived AI Enjoyment (PE) and Perceived AI Intelligence (PAI): Survey results supported this hypothesis and are also widely accepted in the literature as well as [89,90]. It showed that enjoyment shapes educators' opinions of AI's intelligence. The interviews revealed that educators who enjoyed using AI technology saw it as smarter. “Several participants described their interactions with ChatGPT as an engaging and enjoyable learning experience. They found the AI tool interactive, stimulating their curiosity and interest in the subject”. Lesson plan reviews showed that educators who used AI to engage students thought it was smarter. Perceived AI Enjoyment and Technology Acceptance: This hypothesis was significant but weaker. According to interviews, AI technology was accepted by educators who enjoyed utilizing it [24,89,90]. The lesson plan evaluations showed that educators who used AI technology in fun and engaging ways had greater intentions of using it again. “Some participants perceived enjoyment as linked to positive emotions such as curiosity, satisfaction, and a sense of accomplishment. These emotional responses contributed to a favorable learning environment”.

Perceived AI Intelligence (PAI) and adoption of AI Technology (ATU): The survey data highly supported this hypothesis as previous studies supported this [88]., showing that educators' opinions of AI's intelligence strongly influence their adoption of AI technology. The importance of perceived intellect in acceptance was confirmed in interviews. “Participants recognized the AI's cognitive capabilities in comprehending the context of their inquiries. They highlighted the AI's ability to engage in meaningful conversations, which they associated with higher intelligence”. The lesson plan ratings showed that educators who understood and integrated AI were more receptive of it.

### 5.6 Perceived AI intelligence

Perceived AI Intelligence (PAI) and Metacognitive Self-Regulated Learning (MSR): The survey strongly supported this hypothesis, demonstrating that perceived AI intelligence strongly affects instructors' MSR [95]. In interviews, educators said AI's intelligence helped them encourage self-regulated learning. “Some participants commented on the AI's adaptability and responsiveness to various topics, indicating its intelligence in catering to diverse educational needs”. Lesson plan assessments supported this link, showing that AI technology could promote metacognitive self-regulated learning. Perceived AI Intelligence (PAI) and Behavioral Intention to Use AI (BIU): Survey data did not strongly support this concept. Participants believed that using AI tools they associated with higher intelligence [95]. However, lesson plan assessments showed that educators who found AI's intelligence useful intended to apply it. The survey findings strongly supported this prediction, highlighting the importance of technological adoption in molding educators' metacognitive self-regulated learning. Interviews with educators confirmed that acceptability was key to integrating AI into self-regulated learning practices. Lesson plan evaluations showed that educators who adopted AI technology used it better for metacognitive self-regulated learning.

### 5.7 attitude toward use ChatGPT

Attitude toward use ChatGPT (ATU) and Behavioral Intention to Use AI (BIU): Survey results strongly supported this prediction, showing that educators' attitude of AI positively influenced their intention to use it [110,111]. Interviews stressed the link between attitude and intention. “The majority of participants exhibited a positive attitude toward using ChatGPT for metacognitive self-regulated learning. They expressed enthusiasm for AI-powered tools in education “. And “some participants noted that their experiences with ChatGPT increased their confidence in tackling challenging subjects. They felt more empowered in their learning journey”. Lesson plan evaluations showed that educators who strongly accepted AI technology intended to use it for education.

### 5.8 Metacognitive self-regulated learning

Metacognitive Self-Regulated Learning (MSR) and Behavioral Intention to Use AI (BIU): Survey data strongly supported this hypothesis, showing that metacognitive self-regulated learning strongly influences educators' intentions to use AI for education and support the literature studies and aligned [63,95,99].

### 5.9 Reflective interview questions and record analysis

The post-reflection themes significantly contribute to this narrative, providing a qualitative dimension to educators' perceptions of ChatGPT's support for MSRL across various teaching phases. Notably, educators found ChatGPT beneficial during the Planning phase for lesson content planning, objective setting, strategy outlining, and resource organization. Positive feedback during the Presentation phase emphasized enhancements in lesson clarity, content quality, and alignment with learning objectives. While the majority reported positive experiences, some acknowledged challenges, particularly in tracking progress during the Reflection phase. These reflections align with prior literature, affirming the instrumental role of technology, such as ChatGPT, in supporting diverse dimensions of Metacognitive Self-Regulated Learning (MSRL). The positive experiences reported by educators resonate with broader studies showcasing technology's positive impact on teaching practices and learner outcomes.

The evaluation of lesson plans further highlights the potential of ChatGPT to facilitate Metacognitive Self-Regulated Learning (MSRL). A significant majority of assessed lesson plans exhibited clarity, logical coherence, and alignment with learning outcomes, indicating the positive influence of ChatGPT on the quality of lesson planning. While these findings showcase the positive impact on educators' ability to develop high-quality lesson plans, it's crucial to address specific areas identified for improvement, particularly in consistent alignment with learning outcomes and the inclusion of engaging elements.

The results highlight the multifaceted impact of ChatGPT on educators' attitudes, perceptions, and practices related to MSRL. The positive reflections and lesson plan evaluations corroborate the hypothesis testing results, emphasizing the overall positive influence of ChatGPT on teaching practices and learning outcomes. The nuanced relationships in the survey findings resonate with educators' lived experiences and practical utilization of ChatGPT. These findings align with existing literature emphasizing the importance of perceived competence, social influence, trust, usefulness, and enjoyment in technology acceptance. The positive impact of perceived AI intelligence on attitudes and metacognitive self-regulation reinforces the potential of ChatGPT in enhancing teaching practices and supporting MSRL.

## 6 Theoretical and practical implications of the study

### 6.1 Theoretical implications

Our mixed-method study significantly contributes to the theoretical landscape of AI integration in education. By employing a comprehensive approach encompassing surveys, interviews, and lesson plan evaluations, we unveil multifaceted insights into the acceptance and utilization of AI-powered ChatGPT. The study enriches existing theoretical frameworks by identifying critical factors influencing educators' perceptions, attitudes, and practices related to Metacognitive Self-Regulated Learning (MSRL). The nuanced relationships revealed, particularly the relationship of Personal Competence (PC), Social Influence, Perceived AI Trust, and Enjoyment with perceived AI intelligence and attitudes, deepen our understanding of the changing aspects of AI adoption.

Furthermore, the study advances theoretical discussions on the role of AI tools, such as ChatGPT, in instructional design. The evaluation of lesson plans provides a practical application of theoretical concepts, shedding light on the tool's impact on defining objectives, ensuring coherence with learning outcomes, and guiding educators in designing engaging and effective lessons. The theoretical implications highlight the evolving nature of educators' roles in the era of AI and the need for practicing frameworks that consider the various dimensions of AI integration in pedagogical practices.

### 6.2 Practical implications

From a practical point of view, our study provides valuable discernment for various stakeholders in higher education. Students express a balanced perspective on the integration of ChatGPT, emphasizing the pivotal roles of educators and institutions in managing its presence. This emphasizes the need for ongoing professional development initiatives to equip educators with the skills to navigate the evolving AI landscape effectively.

Identified factors such as Personal Competence, Social Influence, Perceived AI Trust, Usefulness, Enjoyment, and Intelligence provide practical guidance for educators, institutions, and developers looking to enhance the adoption and integration of AI tools. Acknowledging these factors is crucial for optimizing the use of ChatGPT in educational settings aligning its functionalities with the diverse needs of educators and students. The study's insights into AI tools' emotional and cognitive impact contribute practically by highlighting the enjoyable interaction with ChatGPT. Understanding its potential to improve the learning experience by increasing motivation and creativity underscores AI's positive role in creating supportive and engaging learning environments.

## 7 Conclusion

This study examines pre-service teachers' perspectives of AI-based tools, particularly ChatGPT, in relation to metacognitive self-regulated learning in education. AI tools in education could improve pedagogy and student learning. Our study highlights the key role of ChatGPT in enhancing metacognitive self-regulated learning (MSRL) among pre-service teachers. Our study aimed to explore pre-service teachers' perceptions and use of ChatGPT, which had not been thoroughly examined before. Using a mixed-methods approach, we gathered insights from pre-service teachers. Based on the Technology Acceptance Model (TAM), the quantitative phase revealed that pre-service teachers highly accepted ChatGPT as a tool supporting MSRL in teaching. Individual competencies, social influence, and various AI-related factors were key drivers of this acceptance. The qualitative phase investigated participants' experiences with ChatGPT in scenario-based learning tasks. Their reflections highlighted ChatGPT's positive influence on MSRL, particularly in lesson planning. It also enhanced the quality, clarity, and alignment of the content with learning objectives, as well as content organization, goal setting, and strategy formulation. Lesson plan assessments further validated the effectiveness of ChatGPT in promoting MSRL among pre-service teachers. Ultimately, our research enhances pedagogical practices and student learning outcomes by highlighting the significance and promise of AI technologies such as ChatGPT in teacher education. Based on the data from our study, it can be seen that pre-service teachers in our sample generally accept ChatGPT.

On the other hand, it is impossible to exaggerate the significance of metacognitive self-regulation in the AI integration process. Educators, governing bodies, and institutions have a significant challenge in this study. By emphasizing the requirement for AI competency, the importance of social factors, and the necessity for trust development, it offers useful knowledge that makes integrating AI technologies into teacher preparation programs easier. The triangulation method used in this study also adds a novel and fascinating viewpoint to educational research. Focusing on metacognitive self-regulation, our research advances knowledge of the intricate interactions between AI and pre-service teachers. It provides a remarkable opportunity for teaching and learning, laying the groundwork for further investigation and pointing to the promising possibilities of integrating AI technology into higher education.

## Limitations

There are several limitations to this study. The sample size was limited, consisting of just 300 respondents for the survey and 30 pre-service instructors who participated in interviews. The study would be more generalizable with a larger and more varied sample. The second limitation on the findings' generalizability to other contexts is that the research was done in a particular educational setting. In addition, response bias may have affected the study's primary source of data—self-reported information. There should be objective measures in future studies. Furthermore, there is potential for future research into the long-term consequences of ChatGPT because the study primarily examined the short-term perceptions and effects of the platform.

## Future work

**T**o address these limitations, longitudinal studies evaluating ChatGPT's long-term effects should be explored in future research. Comparative research can also be used to see how ChatGPT compares against other AI tools and pedagogies. Investigate diverse educational environments ranging from subjects to grade levels for a more thorough understanding. A significant area of research is the ethical issues related to AI in education and the best ways to include AI into teacher education programs. Future research should examine how AI affects student results and how it plays a role in professional growth.

## Funding support

This research was supported by Researchers Supporting Project number (RSP2024R159), 10.13039/501100002383King Saud University, Riyadh, Saudi Arabia.

## Informed consent statement

All participants provided informed consent before joining the study.

## Data availability statement

Data will be made available on request to corresponding authors.

## Ethical approval statement

The research study mentioned above involved the collection of data from UTM Malaysia, and prior ethical approval was duly obtained, under Reference No. UTM.J.13.01/13.14/1/88 Jld.23 (75)/Dated: 1-06-2023 and under RMC research project no. Q.J130000.21A2.07E10.

## CRediT authorship contribution statement

**Nisar Ahmed Dahri:** Writing – original draft, Software, Formal analysis, Data curation, Conceptualization. **Noraffandy Yahaya:** Supervision, Investigation. **Waleed Mugahed Al-Rahmi:** Writing – original draft, Validation, Supervision, Methodology. **Ahmed Aldraiweesh:** Writing – review & editing, Investigation, Funding acquisition, Formal analysis. **Uthman Alturki:** Writing – review & editing, Validation, Project administration, Funding acquisition. **Sultan Almutairy:** Validation, Project administration, Methodology, Data curation. **Anna Shutaleva:** Visualization, Software, Methodology, Formal analysis, Data curation. **Rahim Bux Soomro:** Writing – review & editing, Validation, Formal analysis, Conceptualization.

## Declaration of competing interest

The authors declare that they have no known competing financial interests or personal relationships that could have appeared to influence the work reported in this paper.
