# Supplementary material for: Extended TAM based acceptance of AI-Powered ChatGPT for supporting metacognitive self-regulated learning in education: A mixed-methods study
Source: Heliyon. 2024 Apr 9;10(8):e29317. doi: 10.1016/j.heliyon.2024.e29317 (PMC11016976; doi:10.1016/j.heliyon.2024.e29317)
Supplement: Multimedia component 1 [file mmc1.docx]

**Appendix A. Scenario based learning Tasks**

| **MSRL Strategy** | **Scenario No** | **Scenario Name** | **Summary** |
| --- | --- | --- | --- |
| Activation of prior content knowledge | S01 | ChatGPT Lesson Recall Assistant | ChatGPT assists pre-service teachers in recalling prior lesson content relevant to their current lesson plan creation. |
| Setting goals | S02 | ChatGPT Learning Objective Setter | ChatGPT helps pre-service teachers set clear learning objectives for their lesson plans and suggests action plans. |
| Activation of task value and interest | S03 | ChatGPT Engagement Enhancer | ChatGPT provides engaging content ideas and methods to keep students interested in the lesson plans being created. |
| Selection and adaptation of cognitive strategies | S04 | ChatGPT Resource Recommender | ChatGPT recommends suitable study resources, materials, and strategies for pre-service teachers' lesson plans based on their individual needs and preferences. |
| Metacognitive monitoring | S05 | ChatGPT Progress Tracker | ChatGPT monitors pre-service teachers' progress in creating lesson plans, providing feedback and suggestions for improvement. |
| Selection strategies for managing motivation and affect | S06 | ChatGPT Motivation Booster | ChatGPT offers motivational content and insights to keep pre-service teachers motivated and enthusiastic while creating lesson plans. |
| Help-seeking behavior | S07 | ChatGPT Help Desk Assistant | ChatGPT answers questions and provides guidance to pre-service teachers in real-time as they work on their lesson plans. |
| Reviewing | S08 | ChatGPT Review and Feedback | ChatGPT allows pre-service teachers to review and revise their lesson plans, offering feedback and recommendations based on best practices. |
| Self-evaluation | S09 | ChatGPT Self-Assessment Aid | ChatGPT generates self-assessment quizzes for pre-service teachers to evaluate the quality and effectiveness of their lesson plans. |
| Self-satisfaction | S10 | ChatGPT Self-Recognition | ChatGPT helps pre-service teachers recognize their achievements and improvements in lesson plan creation, reinforcing their self-confidence |

**Appendix B. Structured Questionnaire**

**Section A: Demographic Information- Tells us about yourself.**

| Name (Optional) | |  | | | |
| --- | --- | --- | --- | --- | --- |
| Age group | 20-30 (1) | 31-40 (2) | 41-50 (3) | 51-60 | Other (5) |
| i) Gender of the respondent | | Male 🞐 Female 🞐 | | | |
| ii) Have you used AI for teaching and learning? | | Yes 🞐 No 🞐 | | | |
| iii) Do you have a knowledge of ChatGPT? | | Yes 🞐 No 🞐 | | | |

**Section B: AI Adoption Tool**

*Please rate the extent to which you agree with each statement below.*

(Please check √ the most appropriate option for each statement below)

**1=Strongly Disagree (SD) 2= Disagree (D) 3= Neutral(N) 4= Agree(A) 5= Strongly agree (SA)**

| **Personal Competence (5 items)** | | **SD** | **D** | **N** | **A** | **SA** |
| --- | --- | --- | --- | --- | --- | --- |
| PC1 | I believe I possess the skills required to effectively use ChatGPT for learning |  |  |  |  |  |
| PC2 | I feel confident in my ability to navigate and utilize ChatGPT for metacognitive self-regulated learning. |  |  |  |  |  |
| PC3 | I am proficient in adapting ChatGPT to enhance my learning experience. |  |  |  |  |  |
| PC4 | I have the necessary knowledge and competence to make the most of ChatGPT for learning. |  |  |  |  |  |
| PC5 | My level of competence enables me to use ChatGPT productively for metacognitive self-regulated learning. |  |  |  |  |  |
| **Social Influence (5 items):** | | **SD** | **D** | **N** | **A** | **SA** |
| SI1 | The opinions of my peers influenced my decision to use ChatGPT for learning. |  |  |  |  |  |
| SI2 | I consider the recommendations of my instructors when deciding to use ChatGPT. |  |  |  |  |  |
| SI3 | The guidance from educational experts plays a significant role in my choice to use ChatGPT for metacognitive self-regulated learning. |  |  |  |  |  |
| SI4 | My friends' perceptions of ChatGPT impact my acceptance and utilization. |  |  |  |  |  |
| SI5 | I am influenced by the experiences and feedback of others in my educational community regarding ChatGPT. |  |  |  |  |  |
| **Perceived AI Trust (4 items):** | | **SD** | **D** | **N** | **A** | **SA** |
| PT1 | I trust ChatGPT to provide reliable and accurate information for my learning. |  |  |  |  |  |
| PT2 | I have confidence in the capabilities of ChatGPT for assisting in my metacognitive self-regulated learning. |  |  |  |  |  |
| PT3 | I believe ChatGPT is a trustworthy tool for enhancing my learning experiences. |  |  |  |  |  |
| PT4 | My trust in ChatGPT contributes to my willingness to use it for metacognitive self-regulated learning. |  |  |  |  |  |
| PT5 | ChatGPT's adaptability to my learning needs boosts my trust in its educational support |  |  |  |  |  |
| **Perceived AI Usefulness (4 items):** | | **SD** | **D** | **N** | **A** | **SA** |
| PU1 | I find ChatGPT useful for improving my metacognitive self-regulated learning. |  |  |  |  |  |
| PU2 | ChatGPT adds value to my learning process. |  |  |  |  |  |
| PU3 | I perceive ChatGPT as a beneficial tool for my educational development. |  |  |  |  |  |
| PU4 | The utility of ChatGPT enhances my overall learning experience. |  |  |  |  |  |
| PU5 | ChatGPT enhances my learning experience. |  |  |  |  |  |
| **Perceived AI Enjoyment (5 items):** | | **SD** | **D** | **N** | **A** | **SA** |
| PE1 | I enjoy using ChatGPT for metacognitive self-regulated learning. |  |  |  |  |  |
| PE2 | Interacting with ChatGPT makes my learning experience more enjoyable. |  |  |  |  |  |
| PE3 | ChatGPT's features and capabilities are enjoyable to use for educational purposes. |  |  |  |  |  |
| PE4 | I derive pleasure from utilizing ChatGPT in my learning activities. |  |  |  |  |  |
| PE5 | My interactions with ChatGPT add a sense of enjoyment to my metacognitive self-regulated learning. |  |  |  |  |  |
| **Perceived AI Intelligence (5 items):** | | **SD** | **D** | **N** | **A** | **SA** |
| PAI1 | I believe ChatGPT demonstrates a high level of intelligence in assisting with my learning. |  |  |  |  |  |
| PAI2 | ChatGPT's problem-solving abilities impress me. |  |  |  |  |  |
| PAI3 | I perceive ChatGPT as highly intelligent in understanding and supporting my learning needs. |  |  |  |  |  |
| PAI4 | ChatGPT's intelligence significantly contributes to its effectiveness in supporting my learning. |  |  |  |  |  |
| **Attitude toward use ChatGPT (5 items):** | | **SD** | **D** | **N** | **A** | **SA** |
| ATU1 | I have a positive attitude toward using ChatGPT for metacognitive self-regulated learning. |  |  |  |  |  |
| ATU2 | My overall perception of ChatGPT is favorable for enhancing my learning. |  |  |  |  |  |
| ATU3 | I hold a constructive attitude regarding ChatGPT's impact on my educational development. |  |  |  |  |  |
| ATU4 | My attitude toward using ChatGPT is optimistic and encouraging. |  |  |  |  |  |
| **Metacognitive Self-regulation Learning (4 items):** | | **SD** | **D** | **N** | **A** | **SA** |
| MSR1 | I effectively employ metacognitive strategies when using ChatGPT for learning. |  |  |  |  |  |
| MSR2 | ChatGPT enhances my ability to regulate my learning processes effectively. |  |  |  |  |  |
| MSR3 | I am proficient in applying metacognitive skills while using ChatGPT. |  |  |  |  |  |
| MSR4 | ChatGPT supports my metacognitive self-regulated learning practices. |  |  |  |  |  |
| **Intention to use ChatGPT (4 items):** | | **SD** | **D** | **N** | **A** | **SA** |
| BIU1 | I have a strong intention to continue using ChatGPT for metacognitive self-regulated learning. |  |  |  |  |  |
| BIU2 | I am determined to incorporate ChatGPT into my learning routines. |  |  |  |  |  |
| BIU3 | My intention is to regularly use ChatGPT as a valuable learning tool. |  |  |  |  |  |
| BIU4 | I plan to consistently utilize ChatGPT for metacognitive self-regulated learning. |  |  |  |  |  |

**Appendix C. Post-Task Reflection Tool**

| The post-task reflection tool is designed to gather qualitative insights from participants' experiences. It consists of open-ended questions that prompt participants to reflect on their interactions with ChatGPT and metacognitive self-regulated learning experiences. | |
| --- | --- |
| **1** | Please describe your experience using ChatGPT for metacognitive self-regulated learning. |
| **2** | What were the challenges you encountered while using ChatGPT, if any? |
| **3** | Share any positive aspects of your experience with ChatGPT. |
| **4** | How do you perceive the impact of ChatGPT on your metacognitive self-regulated learning? |
| **5** | Were there any specific instances where ChatGPT significantly contributed to your learning? Please provide details. |

**Appendix D. Lesson Plan Evaluation Checklist**

| **SNO** | **Statement** | **Tick 🗸** | |
| --- | --- | --- | --- |
|  |  | **Yes** | **NO** |
| **1** | Clarity of Objectives/ Lesson objectives |  |  |
| **2** | Content Coherence / Subject matter knowledge |  |  |
| **3** | Alignment with Learning Outcomes |  |  |
| **4** | Engagement Strategies /Lesson activities |  |  |
| **5** | Assessment of the lesson |  |  |
